# Lunacy Work in London and in New York

**Published:** 1901-09-21

**Authors:** 


### LUNACY WORK IN LONDON AND IN NEW YORK.

THE County of London has to look after about 20,000 lunatics, and it adds something like 500 a year to its insane population as accommodated in the public asylums. The cost of maintenance of each lunatic therein is not less than £25 a year; and even this sum does not include the interest on capital spent in building the asylums, nor in keeping them in order. We read of £114,000 having been spent in the erection of one asylum, and another is supposed to have cost four times as much as that. We hear of £2,900 being spent at one asylum in one year on alterations and additions, and of £4,000 being similarly spent at another. At Colney Hatch the committee were prepared to lay out £2,000 on an isolation hospital for women—a sum amply sufficient for the purpose one would have thought—but we are told that, when the plans were sent to the office of the Commissioners in Lunacy, amendments were there suggested which "involved such a large expenditure beyond that voted that we have postponed the provision for the present." Are the committee of that asylum prepared to take up a position, and will that position be really effective unless backed up strongly by public opinion, and enforced by some modification of the plan lately inaugurated in New York?

That State is in a worse condition than London, for every year it is adding 700 to its army of the insane, which army already numbers 24,000 people. The expense of housing and maintaining this army has assumed gigantic proportions, and, like our own, it was an increasing one. Some ten years ago the ratepayers took the matter in hand, and those of London may learn a lesson from them. After years of constant work two distinct results have been attained in New York. "These are," the committee say, "economy of administration and the near termination of overcrowding." And it is further pointed out that the old extravagant days have passed away—days when the cost per bed ran up to 2,000 or even 3,000 dollars. Now a State Care Law declares that the *per capita* cost of buildings fully equipped, including heating, lighting, and furniture, should not exceed 550 dollars —say £110.

The American report continues:—"At first there was much friction. The hospitals were accustomed to being extravagant and liked it; they objected to careful scrutiny and cutting down of their estimates by the Commission." Reforms were, however, gradually introduced in the economical management of asylums, and the efforts of the committee have resulted in a lowering of the maintenance rate from 216 dollars per annum in 1893 to 165 dollars in 1900, the difference being about equal to £10 a year on each patient.

Much of what may be described as internal economy has resulted from adopting the "joint contract system"—that is, all staple articles are bought wholesale, and certain manufactured articles are made at one of the asylums and distributed to the others.

We have said enough to show the direction in which the American committee has moved, and the success which it has met with may encourage a movement of some kind in England.

